# Two draft genomes of fungal leaf endophytes from tropical gymnosperms

**DOI:** 10.1128/mra.00511-24

**Published:** 2024-10-02

**Authors:** Juan Carlos Villarreal Aguilar, Omayra Meléndez, Rita Bethancourt, Ariadna Bethancourt, Lilisbeth Rodríguez-Castro, Jorge Mendieta, Armando Durant, Marta Vargas, Brian Sedio, Kristin Saltonstall

**Affiliations:** 1 Smithsonian Tropical Research Institute, Ancón, Panamá; 2 Department of Biology, Université Laval, Québec City, Québec, Canada; 3 Departamento de Microbiología y Parasitología, Universidad de Panamá, Panama City, Panama; 4 Departamento de Botánica, Universidad de Panamá, Panama City, Panamá; 5 Department of Integrative Biology, University of Austin, Texas, USA; University of California Riverside, Riverside, California, USA

**Keywords:** cycad, endophyte, pathogen, ascomycetes, secondary metabolites, *Zamia*

## Abstract

Two ascomycetes, *Neofusicoccum* sp. and *Xylaria* sp.*,* were isolated from healthy leaves of the tropical gymnosperms *Zamia pseudoparasitica* (Z2) and *Zamia nana* (Z50) from Panama. The two draft genomes possess a broad predicted repertoire of carbohydrate-degrading CAZymes, peptidases, and secondary metabolites, with more secondary metabolite clusters in the *Xylaria* isolate.

## ANNOUNCEMENT

*Neofusicoccum* and *Xylaria* are two common endophytic fungi (1, 2) isolated from two endemic cycad species from Panama. Cycads are the most endangered group of plants—nearly 72% of the 375 species have a critical IUCN status. The main threats are deforestation and poaching. To our knowledge, these are the two first fungal genomes isolated from cycads.

The two cultures were sampled from *Zamia pseudoparasitica* (Z2) and *Zamia nana* (Z50) from El Copé (8°40′12.12″N, 80°36′13.26″W) and El Valle de Antón (8°37′18.32.52″N, 80° 7′13.9548″W), respectively, in Central Panamá. Briefly, middle sections of leaf samples were cut into 50 2 × 2 mm^2^ fragments and surface sterilized by placing them in a small strainer that was submerged and shaken constantly while they were passed through a disinfection battery using a 70% ethanol wash for 2 min, 1% sodium hypochlorite for 3 min, and sterile distilled water for 1 min. The fragments were seeded on large Petri dishes (90 × 14 mm) containing solid potato dextrose agar (PDA) and incubated at 24°C–26°C (ambient light) for approximately 1 week to allow fungal growth to emerge. To isolate pure cultures, a fragment of mycelium was taken from each cultivar, transferred to a test tube with inclined PDA, and grown for nearly 2 months using sterile tweezers. Cultures have been deposited in the collection of Department of Microbiology, Universidad de Panama.

Genomic DNA was extracted using a cetyltrimethylammonium bromide (3) method (obtaining up to 120 ng in 11.7 µL). The genomic DNA was used for library synthesis using a KAPA HyperPlus Kit (Roche), according to the manufacturer’s instructions. The library was quantified and sequenced on an Illumina MiSeq 150-bp paired-end run (300 cycles, v2 kit) at the Smithsonian Tropical Research Institute (Panamá). DNA reads were cleaned and trimmed using Trimmomatic version 0.36 (4) (-phred33), read quality was assessed using FastQC version 0.11.8 (5), and *de novo* assembled using SPADes version 3.14.1 (6). Genome quality and coverage were assessed using Minimap 2.1.0 (7). Fungal identity was verified using BUSCO version 5.0.0 (8), BLAST version 2.9.0+ (9), and BlobTools version 1.1 (10). After selecting only ascomycete contigs and verifying their taxonomic identity using BLAST, BUSCO was used to estimate the completeness of the filtered assemblies.

**Fig 1:**
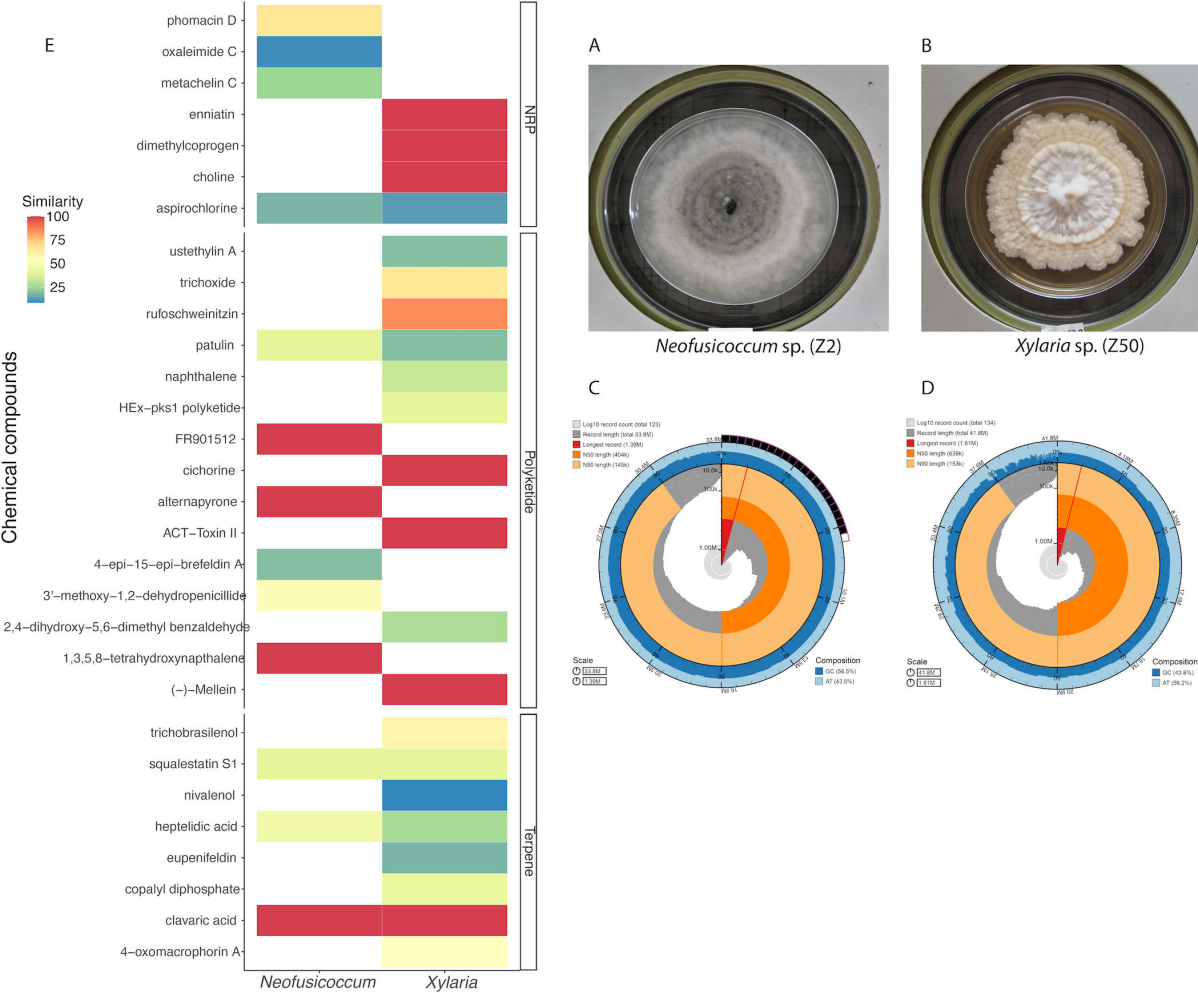
Morphological and genomic features of fungal genomes. **(A)** Culture of *Neofusicoccum* sp. (**Z2**). (B) Culture of *Xylaria* sp. (**Z50**). (C) Snail plot indicating general features of the genomes, such as *N*_50_, scaffold length, and GC content of *Neofusicoccum* sp. (**Z2**). (D) Snail plot indicating general features of the genomes such as *N*_50_, scaffold length, and GC content of *Xylaria* sp. (**Z50**). (E) Secondary metabolite gene clusters (SMGCs) predicted from antiSMASH analyses for both genomes, highlighting non-ribosomal peptides (NRPs), polyketides, and terpenes. A complete annotation of the SMGCs can be found at https://github.com/jcarlosvillarreal/fungal_cycad_genomes_Panama.

We then used the Funannotate version 1.8.12 pipeline (11) to mask repeats, predict, annotate, and compare the genomes. We used the “funannotate predict” command to train and run three *ab initio* gene predictors—AUGUSTUS version 3.3.2 (12), GlimmerHMM version 3.4 (13), and SNAP v2006-07-28 (14). Functional prediction of the gene models was performed using InterProScan version 5.57-90.0 (15) with mapping to Gene Ontology (GO) terms, eggNOG-mapper version 2 (16), the Clusters of Orthologous Groups of proteins (17), Pfam domains, the Carbohydrate-Active Enzyme database [CAZY (18)], the secreted protein database [MEROPSv12 (19)], and InterProScan version 5.57-90.0 (15) for fungal transcription factors. We explored the richness of secondary metabolite gene clusters (SMGCs) using antiSMASH version 6.1.1 (20). The relaxed search was conducted on scaffolds and annotated genes (from the funannotate output “annotate results”) using the online settings knownClusterBlast, ClusterBlast, SubClusterBlast, ActiveSiteFinder, Cluster Pfam analysis, and Pfam-based GO term annotation. The genome statistics for each strain are indicated in Fig. 1; Table 1.

**TABLE 1:** Genome statistics for fungal isolates from *Zamia pseudoparasitica* (Z2) and *Zamia nana* (Z50) from Panama

Parameter	*Neofusicoccum parvum* (Z2)	*Xylaria* sp. (Z50)
No. of clean reads	5,599,532	6,504,418
Total genome size (bp)	33,764,537	41,770,564
Largest scaffold	1,389,236	1,612,932
Number of scaffolds	123	134
*N*_50_ (bp)	403,681	638,724
Coverage (×)	62	64
GC content (%)	56.53	43.83
No. of genes	9,753	10,245
No. of proteins	9,634	10,030
No. of tRNAs	119	215
Completeness (%) (BUSCO)	92,5	84
Number of secondary metabolite gene clusters	50	95
Number of CAZY enzymes	450	446
Number of secreted peptidases	343	344
Accession no.	JBAWJY000000000	JBAWJU00000000
SRA	SRX22736318	SRX22949399
BioSample	SAMN38641487	SAMN38693397

## Data Availability

The Whole Genome Shotgun project has been deposited in GenBank under the accession no. JBAWJY000000000 (*Neofusicoccum* sp. Z2) and JBAWJU00000000 *(Xylaria* sp*. Z50*). The Project number is PRJNA1048497 and the SRA accession numbers for the raw MiSeq data are SRX22736318 (Z2) and SRX22949399 (Z50). Annotated versions of the genomes can be found in the https://doi.org/10.5281/zenodo.12521997 and github: https://github.com/jcarlosvillarreal/fungal_cycad_genomes_Panama.
